# New genetic and morphological evidence suggests a single hoaxer created ‘Piltdown man’

**DOI:** 10.1098/rsos.160328

**Published:** 2016-08-10

**Authors:** Isabelle De Groote, Linus Girdland Flink, Rizwaan Abbas, Silvia M. Bello, Lucia Burgia, Laura Tabitha Buck, Christopher Dean, Alison Freyne, Thomas Higham, Chris G. Jones, Robert Kruszynski, Adrian Lister, Simon A. Parfitt, Matthew M. Skinner, Karolyn Shindler, Chris B. Stringer

**Affiliations:** 1 Research Centre in Evolutionary Anthropology and Palaeoecology, Liverpool John Moores University, Byrom Street, Liverpool L3 3AF, UK; 2 School of Natural Sciences and Psychology, Liverpool John Moores University, Byrom Street, Liverpool L3 3AF, UK; 3 Department of Anthropology, University College London, 14 Taviton Street, London WC1H 0BW, UK; 4 Department of Earth Sciences, Natural History Museum, London SW7 5BD, UK; 5 Core Research Laboratories, Natural History Museum, London SW7 5BD, UK; 6 Scientific and Library Associate, Natural History Museum, London SW7 5BD, UK; 7 Science Section, Conservation Department, Victoria and Albert Museum, South Kensington, London SW7 2RL, UK; 8 Christopher Ingold Laboratories, University College London, Gordon Street, London WC1H 0AJ, UK; 9 Division of Biological Anthropology, University of Cambridge, Pembroke Street, Cambridge CB2 3QG, UK; 10 Department of Cell and Developmental Biology, University College London, Gower Street, London WC1E 6BT, UK; 11 Oxford Radiocarbon Accelerator Unit, Research Laboratory for Archaeology and the History of Art, University of Oxford, Oxford OX1 3QY, UK; 12 Institute of Archaeology, University College London, London WC1H 0PY, UK; 13 School of Anthropology and Conservation, University of Kent, Marlowe Building, Canterbury CT2 7NR, UK; 14 Department of Human Evolution, Max Planck Institute for Evolutionary Anthropology, Deutsche Platz 6, Leipzig 04103, Germany

**Keywords:** *Eoanthropus*, human evolution, geometric morphometrics, DNA

## Abstract

In 1912, palaeontologist Arthur Smith Woodward and amateur antiquarian and solicitor Charles Dawson announced the discovery of a fossil that supposedly provided a link between apes and humans: *Eoanthropus dawsoni* (Dawson's dawn man). The publication generated huge interest from scientists and the general public. However, ‘Piltdown man's’ initial celebrity has long been overshadowed by its subsequent infamy as one of the most famous scientific frauds in history. Our re-evaluation of the Piltdown fossils using the latest scientific methods (DNA analyses, high-precision measurements, spectroscopy and virtual anthropology) shows that it is highly likely that a single orang-utan specimen and at least two human specimens were used to create the fake fossils. The *modus operandi* was found consistent throughout the assemblage (specimens are stained brown, loaded with gravel fragments and restored using filling materials), linking all specimens from the Piltdown I and Piltdown II sites to a single forger—Charles Dawson. Whether Dawson acted alone is uncertain, but his hunger for acclaim may have driven him to risk his reputation and misdirect the course of anthropology for decades. The Piltdown hoax stands as a cautionary tale to scientists not to be led by preconceived ideas, but to use scientific integrity and rigour in the face of novel discoveries.

## 1. Introduction

In December 1912, palaeontologist Arthur Smith Woodward, Keeper of Geology at the British Museum (Natural History; now the Natural History Museum), and amateur antiquarian and solicitor Charles Dawson announced the sensational discovery of a new fossil hominin: *Eoanthropus dawsoni* (Dawson's dawn man’), otherwise known as ‘Piltdown man’. Dawson first told Smith Woodward of his apparent find in a letter of February 1912, stating that he had found a ‘thick portion of a human(?)[sic] skull which will rival *H. heidelbergensis* in solidity’ [1]. The type specimen of *Homo heidelbergensis* had been discovered in Germany in 1907. The Piltdown find would raise not only the reputation of Dawson and Smith Woodward, but also of Britain as a key nation in the story of human evolution.

**Figure 1.**
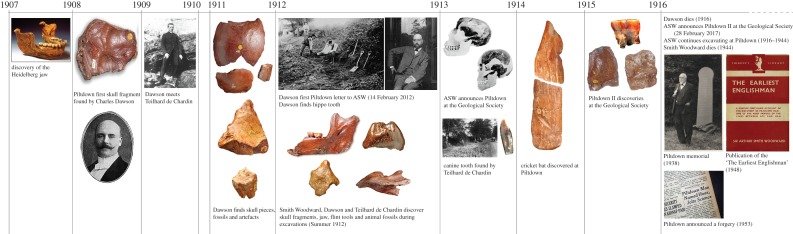
Timeline with the main events and discoveries in the Piltdown history.

The material presented to the Geological Society of London in 1912 consisted of an ape-like mandible containing two worn molar teeth and parts of a human-like braincase, supposedly recovered from a gravel deposit near the village of Piltdown, Sussex, UK. Associated with these were primitive stone tools and fragmentary fossil mammals, all stained dark reddish-brown like the gravels, suggesting an Early Pleistocene or Pliocene date for *Eoanthropus*. Excavations continued in 1913–1914, with the recovery of more artefacts and fauna, and an important addition to *Eoanthropus* in the form of a canine tooth intermediate in size between those of apes and humans. The last significant find at this first Piltdown site (henceforth, Piltdown I) was a remarkable carved slab of bone, which, because of its shape, immediately became known as the ‘cricket bat’ (figure 1). Further work was disrupted by the onset of World War I, and Dawson's declining health and death in August 1916. However, Dawson wrote two postcards to Smith Woodward in 1915 informing him that he had found further faunal and *Eoanthropus* remains at a second site (henceforth, Piltdown II; figure 1), apparently about 3 km from Piltdown I.

The announcement of *Eoanthropus* generated great interest and excitement, both for researchers in relevant fields and the general public alike; however, Piltdown man's initial celebrity has long been overshadowed by its subsequent infamy as one of the most famous scientific frauds in history.

During the early twentieth century, casts (the originals were generally kept locked away and only shown to a select few) of *Eoanthropus* were studied by numerous anatomists (e.g. Keith, Pycraft) [2], and the finds passed into scientific orthodoxy. It then took about 40 years before the elaborate hoax was publicly uncovered [3]. There were early doubters who suggested that a more ancient ape fossil could have become mixed into deposits containing a more recent human cranium [4–6]. They suggested that this view was supported by the mixture of distinct Pliocene and Pleistocene elements in the mammalian assemblage, and the disparity in the technological sophistication of the supposed artefacts. Dawson's subsequent ‘discovery’ of parts of a second Piltdown human at Piltdown II consisting of a tooth and skull fragments matching those from Piltdown I won over some of the dissenters. During the course of the 1920s and 1930s, *Eoanthropus* became increasingly marginalized as ancient hominin fossils were discovered in China, Indonesia and Africa, none of which showed the peculiar combination of an ape-like jaw and human-like braincase found in ‘Piltdown man’.

Increasingly suspicious of the authenticity of *Eoanthropus*, Kenneth Oakley, then Head of Anthropology at the British Museum (Natural History), analysed the portions of Piltdown I and showed that the remains were unlikely to be contemporaneous, and that the jaw was far more recent than suggested by Dawson and Smith Woodward [7–9]. Oxford scientists Joseph Weiner and Wilfrid Le Gros Clark shared his suspicions, and Oakley joined them in more comprehensive studies of the material, published in 1953 and 1955 [3,10]. These showed that a recent ape jaw and canine had been artificially modified, stained and planted at Piltdown I, along with parts of a similarly stained recent human skull. It appeared that the (genuine) mammalian fossils had been gathered from many different localities before being planted in the Piltdown gravels, and the stone tools were similarly introduced, after being artificially stained to match the colour of the gravels. The ‘cricket bat’ was as bogus as the rest—it had been carved with a steel knife, possibly from a fossil elephant bone.

In spite of a series of archival and scientific investigations to resolve the hoax [10–15], our current understanding of how, why and by whom it was designed and perpetrated, is still hampered by various uncertainties. A number of main suspects remain: Charles Dawson, the discoverer of the material and a now-known forger of other specimens; Sir Arthur Smith Woodward FRS, Keeper of the Department of Geology at the Natural History Museum (then the British Museum (Natural History)), friend of Dawson and co-author of the announcement of *Eoanthropus*; Teilhard de Chardin, French Jesuit priest present at some of the excavations and discoverer of the canine; and Martin Hinton, then a volunteer in Woodward's department, who was sceptical of the Piltdown finds. Hinton's experiments with bone and tooth staining were discovered after his death in a chest in the Museum and in his personal effects at Bristol [13]. Understanding what was used to fake the fossil that misled scientists for four decades and how they were manufactured may bring us closer to identifying whether there were one or more hoaxers, and why they would have risked their reputation to fool the scientific community.

Solving the Piltdown hoax is still important now; it stands as a cautionary tale to scientists not to see what they want to see, but to remain objective and to subject even their own findings to the strongest scientific scrutiny. The study of Piltdown man presented here uses scientific methods not available in the earlier twentieth century: DNA analyses, high-precision measurements, spectroscopy and virtual anthropology (study of three-dimensional representations of the specimens) in the hope of finding the answers that have eluded previous investigators. To focus our research, we identified the following series of questions that, if resolved, would narrow down the number of plausible scenarios for the forgery and might help determine the identity of the forger(s):

(Q1) Lowenstein [16] showed that the mandible was likely to have come from an orang-utan (*Pongo* sp.). Are the ape jaw, isolated canine (both Piltdown I) and molar (Piltdown II) indeed from an orang-utan? If so, are they likely to originate from the same animal?
(Q2) How many crania were used to produce the various fragments found at the Piltdown sites and can we assign them to a putative source population?
(Q3) Is there consistency in the *modus operandi* (MO) used to modify the various materials, linking them to one or more forgers?

## 2. Material and methods

All the Piltdown I and Piltdown II materials were available for study (table 1). The methods are described briefly below following the order in which the results are presented; see the electronic supplementary material for further details.

**Table 1.** Piltdown specimens with accession numbers including the original NHM register for the Piltdown II molar.

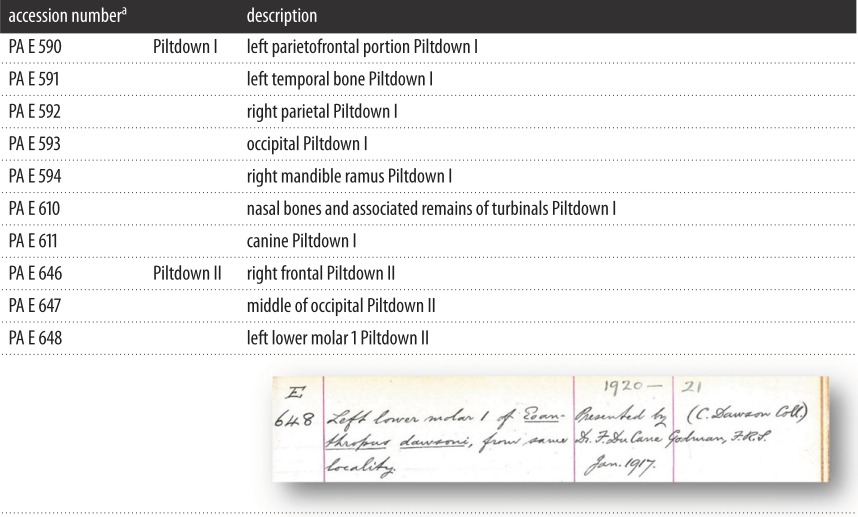

^a^Included in this study.

### 2.1. Morphometric analyses

Linear measurements were taken on the actual Piltdown specimens, and radiographs and μCT scans were compared with data collected on other specimens available for study. See the electronic supplementary material for measurement definitions, μCT scanning protocols and details of the comparative samples.

μCT scans were also used for the geometric–morphometric analysis of the enamel–dentine junction (EDJ) of the three Piltdown molars. After segmentation of enamel and dentine tissues, the surface of the EDJ was produced as a digital surface model. Following methods outlined in the electronic supplementary material and in Skinner *et al*. [17,18], three-dimensional landmarks were used to capture the shape of the EDJ and compare it with a sample of extant apes.

### 2.2. DNA analyses

Sample preparation, DNA extraction and PCR set-up were carried out in a dedicated aDNA laboratory at the Natural History Museum, London (NHM). PCR, qPCR and post-PCR work as well as DNA sequencing were carried out at the NHM, London. The DNA was extracted using a modified silica binding method [19], and PCR was amplified using primers specific to orang-utan, chimpanzee, gorilla or humans (electronic supplementary material, table S5). In order to infer robust consensus sequences and to monitor for contamination and miscoding lesions, we pooled the PCR products into equimolar ratios and sequenced them at approximately 1% of a full Illumina MiSeq lane. Consensus DNA sequences have been uploaded to NCBI GenBank (KX533938-KX533939). See the electronic supplementary material for full description of methods.

### 2.3. Dating analyses

The Piltdown material has been subjected to radiocarbon dating several times, and we hoped to clarify two issues in particular through further dating work at the Oxford Radiocarbon Accelerator Unit. One issue concerns the age of the orang-utan mandible E594, and whether it might have represented a curated ethnographic specimen (e.g. an aboriginal Dayak hunter's ‘trophy head’). A second issue was the question of how many different human specimens were used in the Piltdown I and II forgeries. Dating methods are further described in the electronic supplementary material.

#### 2.3.1. *Modus operandi* investigation

In addition to light microscopy and careful observation of the μCT scans and radiographs, high magnification images of surface modifications on the teeth were recorded using a focus variation microscope (FVM). The FVM used was an Alicona infinite focus optical surface measurement system [20,21] situated at the NHM, which was used to produce three-dimensional micromorphological models to observe any surface modifications.

The XRF analysis of the materials used for staining and filling was performed on an Bruker ArtTAX XRF spectrometer (molybdenum source, 50 kV, 600 µA, lifetime 100 s) housed at the Victoria and Albert Museum with additional analyses on a Bruker Handheld XRF Tracer III SD (max voltage 40 kV) at the Natural History Museum.

## 3. Results

### 3.1. The ape material

**Figure 2.**
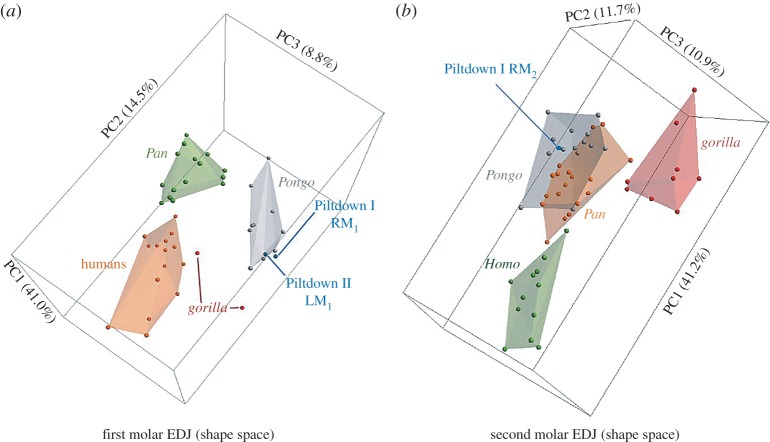
Principal component analysis of first (*a*) and second (*b*) molar EDJ shape.

The first set of analyses focused on the verification and identification of the ape material used in the forgery. Unfortunately, metric evidence based on traditional mandibular measurements (not shown) is inconclusive regarding the taxonomic identity of the Piltdown I mandible, as larger male chimpanzee specimens overlap with those of smaller female orang-utans [22] and the Piltdown specimen is small for an orang-utan. Therefore, we used new methods to examine whether the molars of the Piltdown I mandible can be identified as orang-utan and to assess the likelihood that they derive from the same individual as the Piltdown II molar. Specifically, we conducted a geometric–morphometric analysis of the EDJ on μCT scans of the Piltdown I and Piltdown II molars, as the EDJ has been shown to carry a strong taxonomic signal. It can also classify molars to each taxon (at or above the subspecies level) and molar position [17,18], and is unaffected by any enamel that may have been removed by the forger(s). Figure 2 presents the principal component analyses of M_1_ and M_2_ EDJ shape; in both cases, the Piltdown molars fall within, or in closest proximity to, the *Pongo* sample. Cross-validated canonical variate analyses also consistently classify all three molars as *Pongo* (see the electronic supplementary material). The EDJ of the Piltdown I M_1_ and M_2_ and the Piltdown II M_2_ are similar to *Pongo* in the relative height and spacing of the dentine horns, relative crown height and cervix shape, as well as in the presence of distinctive orang-utan-like wrinkling of the EDJ in the occlusal basins of each molar (see the electronic supplementary material). These results also support the classification of the Piltdown II molar as an M_1_ (see also additional data on pulp chamber similarities between the molars in the electronic supplementary material) and the assertion that the two M_1_s are consistent with being antimeres from the same individual.

**Figure 3.**
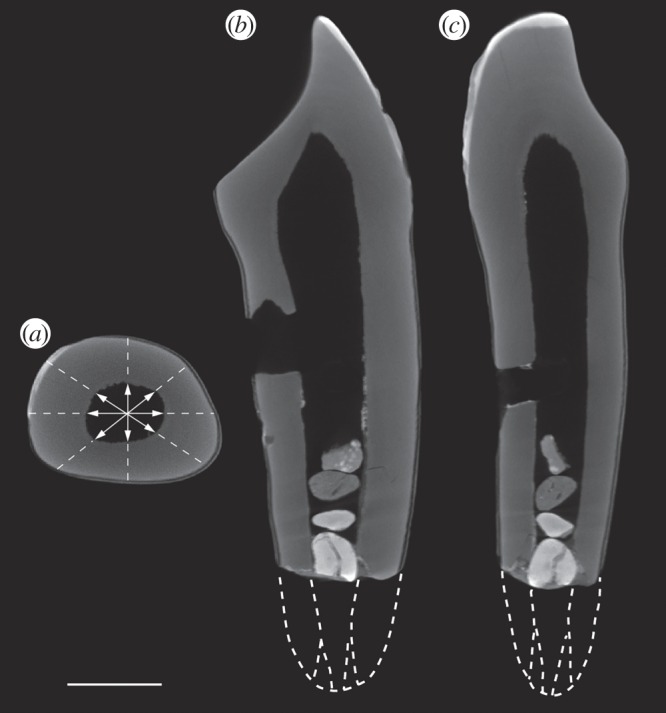
Three μCT scans of the Piltdown canine (scale bar, 5 mm): the transverse scan through the root (*a*) demonstrates enamel just visible to the left (arrows define the diameter of the pulp cavity in four directions) that was compared with that of other apes. The longitudinal section (*b*) in the buccolingual plane shows complete loss of occlusal enamel. In the longitudinal section orthogonal to (*b*) the dotted white lines at the root apex give an indication of how long the undamaged root might have originally been and are reconstructions based on projecting the root contours to follow those typical non-human great ape permanent canines. (*c*) Two possible stages of apex formation are indicated: apex half closed (where the apical walls still diverge from one another) and apex closed with the apical walls parallel.

To discern whether the Piltdown I canine can shed light on the number of individuals used in constructing the non-human parts of the fraud, we employed traditional morphometric methods and radiographs. The crown base shape indices of the Piltdown I canine clearly plot among the mandibular canines and fall outside the range of values for all maxillary canines (the electronic supplementary material); this refutes the contrary ideas of Miller and others [5,23–25], and supports Dawson and Smith Woodward's statements. To assess whether the canine and mandible originated from the same specimen as the mandible, we compared root and socket morphology in permanent canines in a range of great apes of varying ages using standardized radiographs (the electronic supplementary material). The Piltdown I canine is damaged at the root apex and 5–6 mm of root has been lost, making root closure impossible to use as an ageing method (figure 3). Therefore, the remaining root morphology was assessed with regard to its general degree of maturity and the ratio of pulp chamber width at the cervix to cervical root diameter, compared with other great ape specimens. The Piltdown I canine most closely matches those of two subadult orang-utans, which display open root apices for both the M_3_ (as the Piltdown mandible shows) and the permanent canine (figure 3). Thus, these results show that the canine originated from a subadult orang-utan and are consistent with it having originated from the Piltdown I mandible.

**Figure 4.**
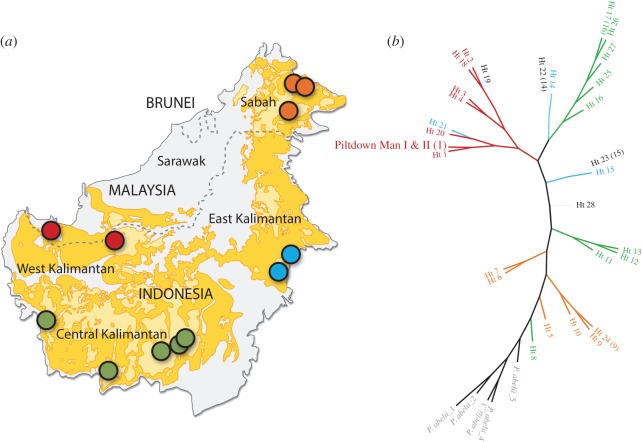
Distribution map (*a*) and phylogenetic tree (*b*) of the orang-utan mtDNA sequences.

The DNA extractions were attempted on the specimens of Piltdown I and Piltdown II, resulting in successfully amplified and sequenced 127 bp mitochondrial DNA in two overlapping fragments from the Piltdown I canine and Piltdown II molar. Unfortunately, we failed to amplify DNA from the Piltdown I mandible. Sequence analysis shows that the Piltdown I canine and Piltdown II molar, however, carried the same mitochondrial haplotype (table 1), consistent with them having originated from a single individual. These results are also consistent with Lowenstein's [16] finding (based on protein electrophoresis) that the specimens are from an orang-utan. Phylogenetic analysis reveals that the Piltdown I canine and Piltdown II molar originated from a Bornean orang-utan (*Pongo pygmaeus*) rather than a Sumatran orang-utan (*Pongo abelii*). Phylogeographic structure of modern populations suggests that the mtDNA haplotype carried by the orang-utan used to forge Piltdown specimens is likely to have originated in west Borneo and, more specifically, in southwest Sarawak (figure 4). However, we caveat this by emphasizing that Bornean orang-utans have suffered from habitat loss and range fragmentation, two processes that can result in rapid shifts in the geographical distribution of genetic lineages [26] (figure 4).

In combination, the geometric morphometric analyses link the Piltdown I mandible and Piltdown II molar; traditional morphometrics link the mandible with the canine, and ancient DNA analysis links the canine and Piltdown II molar. Therefore, given the nature of the context, we consider it highly likely that the Piltdown hoaxer(s) used a single orang-utan specimen originating from southwest Sarawak to construct parts of both Piltdown I and II.

### 3.2. The human material

The second set of analyses focused on the verification and identification of the human material used in the forgery. The cranial bones are all stained brown to match the other specimens. The number of individuals based on cranial fragment preservation confirms that a minimum of two individuals must have been used in the creation of Piltdown I and Piltdown II. Although the thickness of the vault bones was believed to support their prehistoric date and was used by Bowden [27] to exonerate Dawson, it is now known that pathology in modern human vault bones can cause similar thickening [28] or may be part of normal variation.

We attempted to extract DNA from the human remains in order to identify their provenance. Unfortunately, qPCR show that DNA is inadequately, if at all, preserved in the human cranial fragments, with *C*_t_ values consistently higher than our lowest-copy standard reference. Therefore, our DNA analyses are uninformative with regards to how many human individuals were used and their source population.

### 3.3. Radiocarbon accelerator dating

The Piltdown material has been subjected to radiocarbon dating several times, and we hoped to clarify two issues in particular through further dating work. One issue concerns the age of the orang-utan mandible E594, and whether it might have represented a curated ethnographic specimen. This had been suggested by the De Vries and Oakley conventional C14 age determination of 500 ± 100 years, but was contradicted by an Oxford accelerator determination of only 90 (±120), consistent with a Victorian- or Edwardian-age collection [11].

A second issue was whether the Piltdown II frontal bone E646 could be dated to the same age as the Piltdown I cranial fragments, supporting the view that they derived from a single cranium. De Vries and Oakley had obtained an age of 620 (±100) for the Piltdown I E590 parietal, but an Oxford accelerator determination on the E646 frontal [11] provided a date of 970 (±140), which only overlapped at two standard deviations. We sampled all three specimens for further accelerator dating, but the E646 frontal failed chemical pretesting, whereas mandible E594 and parietal E590 showed significant contaminant issues (Tom Higham, personal communication to C. Stringer 2013). In addition, our attempt to date the Piltdown II occipital E647 also failed owing to no yield of dateable material. These failures suggest that the previous conventional and accelerator determinations on the Piltdown orang and human material must be viewed with great caution; and unfortunately, we are unable to make further progress on these issues at present.

### 3.4. The *modus operandi*

The third and final set of analyses focused on determining the MO of the forger(s). The physical specimens, radiographs and high-resolution μCT scans were all carefully studied for signs of modification.

Throughout the Piltdown I and Piltdown II material, a dark reddish-brown stain appears consistent across the assemblage. XRF spectrometry reveals that it is rich in iron and, in some cases, chromium and silver, but it shows little consistency in its actual composition that makes identification difficult. Weiner suggested that a paint similar to Vandyke brown had been applied to the canine [14], but colour variations on the canine and on the rest the assemblage suggest that they may have been made up of different stains available to the forger and these are unidentifiable at present. In addition, the natural deposits at Piltdown are iron-rich, so discerning the stain from the context in which it was deposited has proved difficult. At present, we have been unable to go further than previous studies in determining the exact chemical composition of the staining materials and this will require further analyses.

**Figure 5.**
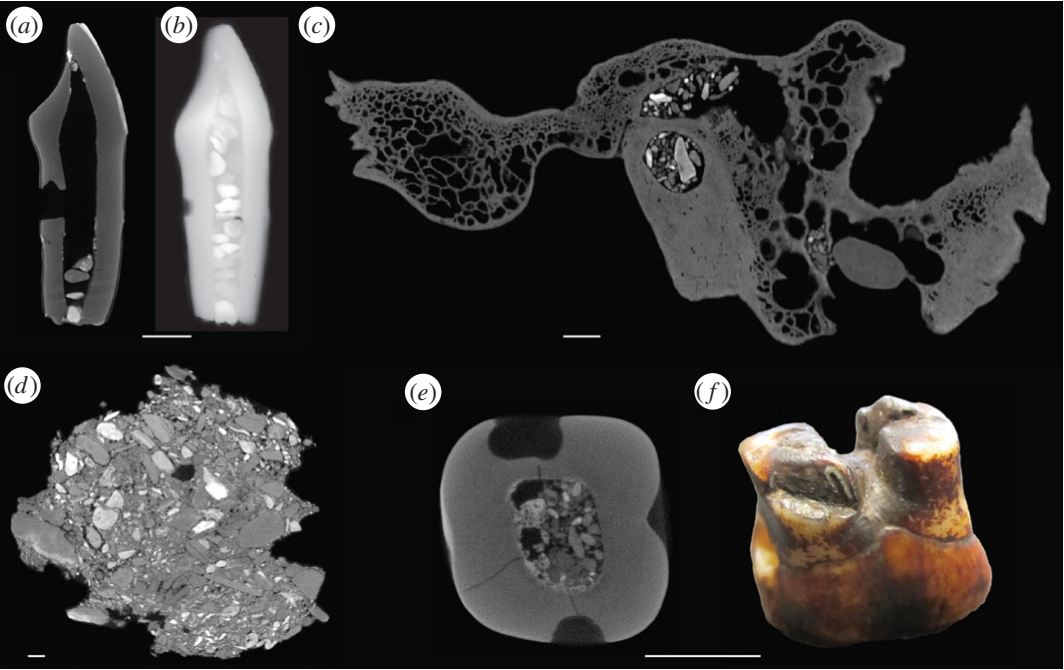
Piltdown gravel is present throughout the Piltdown I and Piltdown II assemblage: µCT scan of the Piltdown I canine (*a*), radiograph from 1925 of the Piltdown canine (*b*) (originally the Piltdown canine had approximately 15 gravel inclusions within the pulp chamber but all but four have now been removed through holes visible in the scan), µCT scan of the Piltdown I temporal with gravel in the cavities and plugged with a pebble in the external acoustic meatus (*c*), µCT scan of the Piltdown gravel (*d*), µCT scan of the Piltdown II molar with gravel in the pulp cavity (*e*), Piltdown II molar with pebbles in the roots (*f*).

Radiographs of the canine taken in 1914 by Reid [29] show gravel inclusions filling the pulp cavity (figure 5; see also [14]). During observations of the μCT scans, inclusions of small gravel particles were noted not only in the canine, but also in the Piltdown I mandible and skull bones, and in the Piltdown II molar. One of the consolidated gravel blocks was also CT-scanned and particles of similar density and size were observed, suggesting that the gravel added to the specimens originated from or around the Piltdown site. These small gravel particles are held in place in a similar manner throughout the specimens. Small pebbles—in elemental signature consistent with the gravel block (the electronic supplementary material)—were used to plug the holes (cranial foramina or dental pulp canals) in the canine, temporal bone and Piltdown II molar (figure 5).

**Figure 6.**
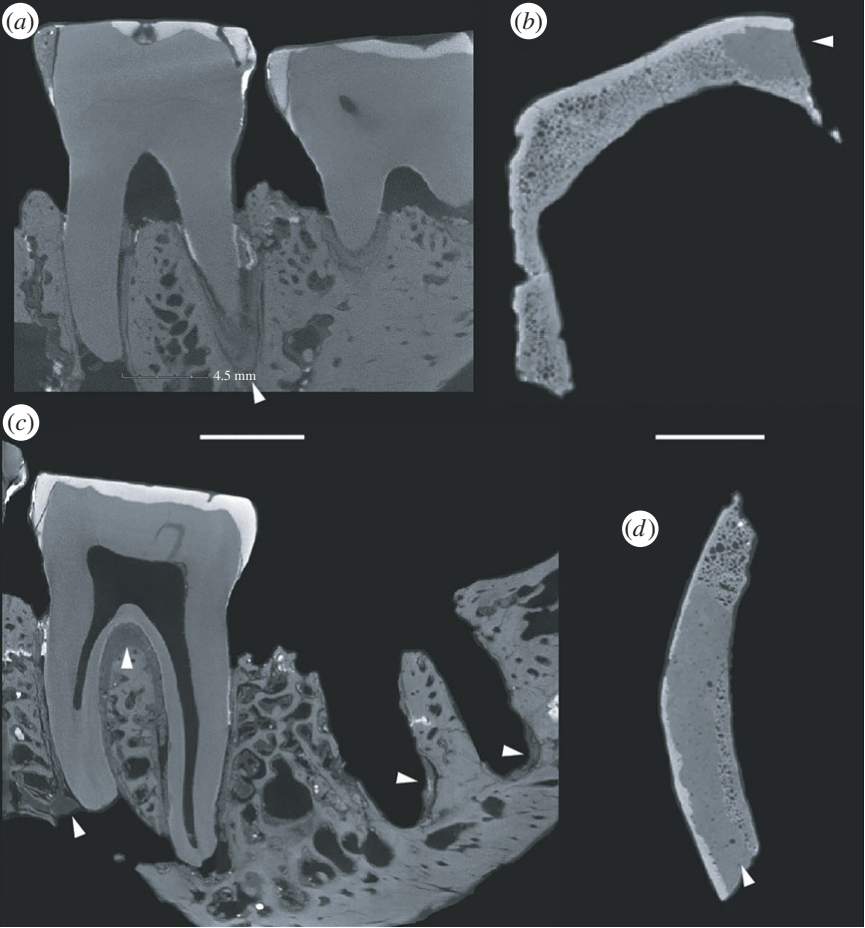
A restorative putty material is present throughout the Piltdown I and Piltdown II assemblage: µCT scan of the heavily restored Piltdown I M_1_ (*a*), µCT scan of the Piltdown I frontoparietal showing putty and overlying radio dense stain (*b*), µCT scan of the Piltdown I mandible with putty present in the periodontal space of the M_2_ and inside the base of the M_3_ cavity (*c*), and the frontal with similar putty where the trabecular bone should be (*d*).

In the temporal bone, the pebble plugs were held in place with a putty containing micro-air pockets and micro-dense inclusions. This putty was discernible in a number of specimens and, where exposed, resembles a white silicate dental filling material when viewed under a binocular microscope. Raman spectrometry was unable to identify the precise material of the putty, but the elemental composition was consistent throughout the measured areas (rich in zinc and zirconium; the electronic supplementary material), matching the location of putty observed in μCT scans across specimens. In the frontoparietal, the putty is observed where there should otherwise be trabecular bone. Earlier interpretations suggested the gypsum observed was of natural origin and owing to the fossilization process of the vault bones [27]. However, in the CT slices, sharp margins between the putty and original bone are observed (figure 6), and the XRF analysis confirms similar elemental properties of this material to the putty that is observed in the heavily fractured first molar in the mandible (figure 6).

**Figure 7.**
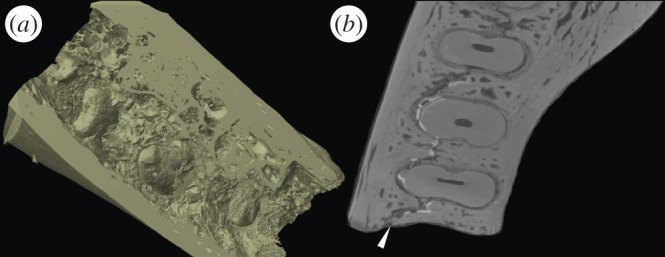
The excavated body of the mandible showing the removed alveolar bone and broken roots (*a*) and crack running lengthwise through the body (*b*).

**Figure 8.**
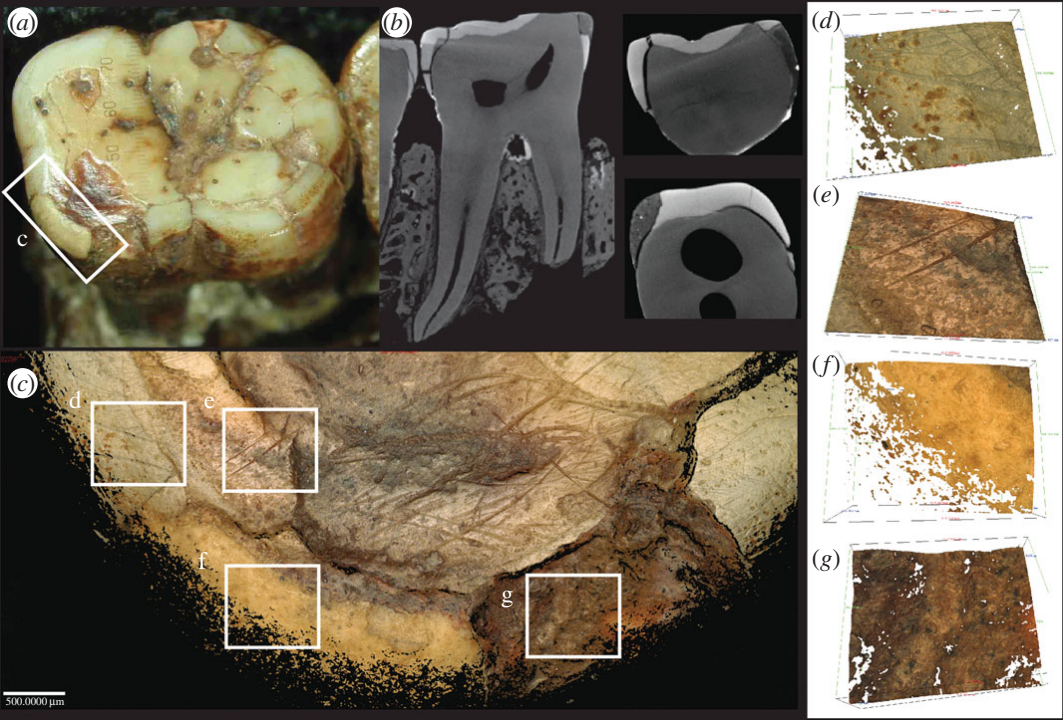
Surface modifications of Piltdown I M_1_: (*a*,*b*) photo and CT scans showing the material removal of the cusps in an unnatural horizontal plane and enamel reconstruction on the lingual margin (*c*) Comparison of surface modifications on different areas of the Piltdown I M_1_: enamel (*d*), dentine (*e*), restorative putty (*f*) and stained enamel (*g*).

Transverse μCT scans through the Piltdown I mandibular body show little or no bony support for the roots cervically, following damage to the alveolar crestal bone. This previously unreported gross internal excavation of the mandibular body beneath the Piltdown I molars, and the crude mechanical removal of alveolar bone above and around the alveolar margins of the mandible, could only have been done if the molar teeth had been removed from their sockets. Transverse μCT scans in the mid-root region also reveal a crack through the alveolar trabecular system running anteroposteriorly along the mandibular body and through the tooth sockets (figure 7). This previously unrecognized crack is likely to have been the (perhaps unintended) result of ‘wish-boning’ the mandible apart at the symphysis while holding it at the condyles, in an attempt to crack and break it across at the symphysis. This would also have had the effect of widening the tooth sockets, making it easier to remove the teeth from the mandible. Great ape molar teeth are very difficult to extract without fracturing their long curved roots. A failed attempt to extract the teeth may have required the fractured root tips be retrieved at any cost, to avoid X-ray detection and resulting in the observed internal excavation. Once removed from the mandible, however, the molar teeth would have been much easier to file and reduce to approximate a more human appearance and pattern of wear. This reduction process was observed closely using the FVM, which indicates that the wear may have been achieved, for example, with abrasives by holding the roots, applying the tooth crown-down on an abrasive surface and subsequently polishing the flattened surface with a rotating rag-mop or with a polishing cloth and rouge or tallow (figure 8). While separate from the mandible, the broken root tips could then have easily been rounded and polished (and stained for a first or second time) to look more like shorter human roots on radiographs.

Both transverse and longitudinal μCT slices through the Piltdown I M_1_, M_2_ and M_3_ socket show further evidence of the same restorative putty present within the periodontal space of the M_1_ and M_2_, and lining the base of the empty M_3_ socket (figure 6). Closer inspection of the Piltdown I M_2_ μCT scans shows this tooth to be improperly seated in its socket, with filling material packed over the socket bifurcation to a thickness of approximately 1.2 mm, more than twice that of a normal periodontal space (figure 6). The filling material in the base of the empty M_3_ socket follows the contours of what might have been the apical root outline of the mesial and distal M_3_ roots. If the mix was too thick and/or the setting time too fast to set the M_1_ and M_2_ in their sockets properly, the forger may have preferred to leave the M_3_ socket empty, rather than replace the tooth incorrectly with respect to the occlusal plane of the M_2_.

## 4. Discussion

The results presented here have added substantial and previously unknown information to the tale of Piltdown man. The orang-utan material used to forge the Piltdown I mandible and molars, and Piltdown II molar very likely originated from a single orang-utan specimen related most closely to the populations now occupying southwest Sarawak (Borneo). Although the historic range of this haplogroup may have varied in the past [26], we can be relatively certain that the orang-utan was born and probably raised in Borneo, since, to the best of our knowledge, no orang-utans successfully produced offspring in Europe until 1926 [30] and most orang-utans brought to Europe around the time Piltdown man was constructed survived on average only a couple of years [31].

The question remains how the orang-utan mandible ended up in the hands of the forger. All of the suspects mentioned in the Introduction would have had access to museum collections and antiquarian shops that may have held these exotic objects after they were collected in the nineteenth to early twentieth centuries which is supported by the dating evidence. Our attempts to find a missing small-sized Bornean orang-utan specimen in the NHM and Hunterian Museum collections have so far not yielded a successful lead.

Two or perhaps three, possibly medieval, humans were used to make up the cranial ‘fossils’. The Piltdown I remains and the Piltdown II frontal were evidently purposely selected for their cranial thickness, whereas the Piltdown II occipital was not. DNA analyses failed to reveal from where these individual specimens originated and dating analyses failed to confirm they come from the same time period, but we have shown that they were subject to the same MO as all of the orang-utan material. Several of the bones and teeth were loaded with gravel that was held in place with pebble plugs, all originating from sediment similar to that found at the Piltdown site. The same putty was used throughout the Piltdown I and Piltdown II assemblages: in order to restore the human vault bones, to hold the gravel plugs in place in the temporal, to restore the M_1_ in the orang-utan mandible and to position the teeth back into place in the mandibular body.

The consistency in the MO observed in the specimens, and the use of a limited number of specimens to create both the Piltdown I and Piltdown II material, are indicative of a single forger. This was most likely Charles Dawson—the prime suspect since the fraud was exposed in 1953 [2]. Over the years, at least 20 others have been accused of being the perpetrator [32], but in many cases, the allegation also includes Dawson as co-conspirator. This is largely because the story originated with him, he brought the first specimens to Dr Arthur Smith Woodward, Keeper of Geology at the British Museum (Natural History) in 1912, nothing was ever found at the site when Dawson was not there, he is the only known person directly associated with the supposed finds at the second Piltdown site, the exact whereabouts of which he never revealed, and no further significant fossils, mammal or human, were discovered in the localities after his death in 1916 [33,34].

Not only did Dawson have the access and connections necessary to obtain the specimens, he was also a great networker, and would have known what the British scientific community was anticipating in a missing link between apes and humans: a large brain, an ape-like face and jaws, and heavily fossilized materials that indicated great antiquity. As a long-established collector, he would also have known what to add in the form of fossil mammals and stone tools to testify to its antiquity. The strategy of the forger(s), in terms of whether the material was sequentially planted at the Piltdown I and Piltdown II sites following a predetermined master-plan or as a response to the reactions engendered by the announcements of the finds, is still unclear (e.g. compare [15,35]), and perhaps both of these factors were at work. For example, the discovery of the canine seems to be a direct response to the vitriolic arguments in the summer of 1913 over the size and shape of Smith Woodward's reconstruction of the skull. At the International Medical Congress on 11 August, Dr Arthur Keith of the Royal College of Surgeons ridiculed it, as *The Times* reported the following day. Just 18 days later, on 30 August, the canine, remarkably similar to the model one Smith Woodward had inserted in the reconstructed jaw, was found and its timely discovery effectively silenced many of the critics. The forged wear on the Piltdown II molar, however, is certainly much more sophisticated than that on the Piltdown I M_1_ and M_2_, and it may be that feedback from discussions after the first finds were announced contributed to this improvement. So many of the hominid bones and teeth from Piltdown I and Piltdown II show a common technical pattern that there seems no reason to suppose that more than one person was responsible for producing them all, even though they may have been crafted over a number of years.

Despite the consistency in production, our novel analyses of the materials from Piltdown have shown that the forger was not a trained conservator. Some aspects of the work show inexpert skills, resulting in bones fracturing, putty setting too fast and teeth cracking while being filed down. Perhaps, if the five years of World War I had not intervened, and more people had been given access to the original material (rather than the casts that were made and offered to other scholars for study), the forgery would have been discovered earlier [2].

The question remains: what would make an apparently successful country solicitor, with a reputation as a geologist, archaeologist and local historian, and with an impressive collection of fossils held in the British Museum (Natural History), turn into a serial forger? If Dawson was the perpetrator of the Piltdown fraud, it has been suggested that his motive was scientific recognition and, in particular, his ambition to be elected a Fellow of the Royal Society [11]. Between 1883 and 1909, Dawson wrote or co-authored more than 50 publications, mainly archaeological or historical and some palaeontological [32,36]. It is an extraordinary list, but none of it appears to have greatly furthered his archaeological or palaeontological career. In 1909, he wrote to Smith Woodward, remarking plaintively, ‘I have been waiting for the big ‘find’ which never seems to come along…’ [37]. Then, in November 1909, Dawson's highly successful younger brother, Arthur Trevor, who was a naval officer and managing director of the defence contractors, Vickers and Son, received a knighthood. It cannot be a coincidence that just six weeks later, Dawson's wife Hélène wrote a remarkable letter to the then Home Secretary, Herbert Gladstone, whom she refers to as an old friend. It was written on 28 December 1909:

My husband, as you will see by enclosed appreciations, has, for a quarter of a century devoted his spare time to scientific labours & has done a great deal for the national collections at the British Museum of Natural History. His services have been absolutely unremunerated & I think you will agree with me that he is entitled to some recognition.

If you think well of it will you be so kind as to commend him for a CB [Companion of the Bath] before the present Government leaves office?’ [38].

Nothing ever came of the letter. Even at this time, Dawson could already have been working on the Piltdown forgery that did at last lead to his nomination for election as a Fellow of the Royal Society in 1913. His nomination was not successful; however, not being elected at the first attempt is not unusual and, had he lived, Dawson may yet have achieved his ambition.

The forgery of Piltdown man fooled many palaeoanthropologists for 40 years, but it has also taught us valuable lessons. We have demonstrated that by meticulous study and the application of new, state-of-the-art scientific techniques it is possible to produce new insights into old palaeoanthropological questions. At the same time, it has opened our eyes to the scientific rigour required to avoid being deceived in the same manner as so many scientists were between 1912 and 1917. As scientists, we must not be led by preconceived ideas in the evaluation of new discoveries. The field of palaeoanthropology is still guilty of fossil hoarding/guarding and exclusivity, but recently, there have been some welcome developments. The treatment of discoveries such as *Homo naledi* in South Africa [39] has demonstrated how rapid dissemination of data and replicas, and free access to publications and to the original specimens can create much greater openness in palaeoanthropology [40]. Such progress should help us avoid the mistakes that the scientific community made when *Eoanthropus dawsoni* was first announced.

## Supplementary Material

De Groote et al. Piltdown SI.

## Supplementary Material

Press statement.docx

## Data accessibility

All DNA sequences are available on NCBI GenBank (KX533938-KX533939). The CT scans can be obtained from the Anthropology Curator at the Natural History Museum, London.
