# Correction to ‘New genetic and morphological evidence suggests a single hoaxer created ‘Piltdown Man’’

**DOI:** 10.1098/rsos.160679

**Published:** 2016-10-12

**Authors:** Isabelle De Groote, Linus Girdland Flink, Rizwaan Abbas, Silvia M. Bello, Lucia Burgio, Laura Tabitha Buck, Christopher Dean, Alison Freyne, Thomas Higham, Chris G. Jones, Robert Kruszynski, Adrian Lister, Simon A. Parfitt, Matthew M. Skinner, Karolyn Shindler, Chris B. Stringer

One of the author names was misspelled in the paper. ‘Lucia Burgia’ should be amended to ‘Lucia Burgio’ and the author list should be as follows:

Isabelle De Groote, Linus Girdland Flink, Rizwaan Abbas, Silvia M. Bello, Lucia Burgio, Laura Tabitha Buck, Christopher Dean, Alison Freyne, Thomas Higham, Chris G. Jones, Robert Kruszynski, Adrian Lister, Simon A. Parfitt, Matthew M. Skinner, Karolyn Shindler and Chris B. Stringer

