# Supplementary material for: New genetic and morphological evidence suggests a single hoaxer created ‘Piltdown man’
Source: R Soc Open Sci. 2016 Aug 10;3(8):160328. doi: 10.1098/rsos.160328 (PMC5108962; doi:10.1098/rsos.160328)
Supplement: De Groote et al. Piltdown SI. [file rsos160328supp1.docx]

# Supplementary Information:

## Micro-CT

All μCT data were collected at the Natural History Museum, London (NHM), using a HMX-ST CT 225 System (Metris X-Tek, Tring, UK). The instrument uses a cone beam projection system [1] with a four megapixel Perkin Elmer XRD 1621 AN3 HS detector panel. μCT data were reconstructed using CT-PRO software version 2.0 (Metris X-Tek) and rendered using VG Studio MAX 2.1 (Volume Graphics, Heidelberg, Germany). Parameters for each specimen are detailed below in Table S1.

Table S1: Micro-CT scan parameters.

| **Specimen** | **Target** | **Filter** | **kV** | **mA** | **Projections / exposure** | **Voxel size (mm)** |
| --- | --- | --- | --- | --- | --- | --- |
| **Piltdown material** |  |  |  |  |  |  |
| Piltdown I canine | W | 0.25 mm Al | 135 | 200 | 3142 / 0.354 s | 0.017 |
| Piltdown I M_1_ & M_2_ | W |  | 145 | 200 | 6284 / 0.345 s | 0.0167 |
| Piltdown II M_1_ | W |  | 135 | 200 | 3142 / 0.354 s | 0.0095 |
| Piltdown I parietal | W |  | 180 | 180 | 3142 / 0.354 s | 0.0842 |
| Piltdown I occipital | W |  | 180 | 171 | 3142 / 0.354 s | 0.0577 |
| Piltdown I temporal | W |  | 180 | 171 | 3142 / 0.354 s | 0.0472 |
| Gravel block | W | 0.5 mm Cu | 150 | 130 | 3142 / 0.354 s | 0.0979 |
| Gravel block | W | 0.5 mm Al | 190-200 | 180-200 | 3142 / 0.354 s | 0.0887-0.1089 |
| **Comparative material** |  |  |  |  |  |  |
| Orang-utan mandible | W | 0.5 mm Cu | 150 | 130 | 3142 / 0.354 s | 0.0979 |
| Molar region orang-utan mandible | W |  | 155 | 165 | 3142 / 0.354 s | 0.0261 |
| Chimpanzee mandible | W | 0.5 mm Ci | 160 | 200 | 3142 / 0.354 s | 0.0238 |

## SI for GMM analyses

**Geometric morphometric comparison of enamel-dentine junction shapes**

**Comparative sample**

The sample comprised μscans of hominoid mandibular molars (see Table S1) from Hull and East Riding Museum, UK; Senckenberg Forschungs Institute und Naturmuseum; Museum für Naturkunde; Max Planck Institute for Evolutionary Anthropology, all Germany; and the Francis J. Rainer Institute of Anthropology, Romania.

Table S1: Comparative sample of mandibular molars for EDJ morphology analyses.

| **Species** | **M_1_ sample** | **M_2_ sample** |
| --- | --- | --- |
| Gorilla | 2 | 9 |
| Chimpanzee | 15 | 18 |
| Orang-utan | 10 | 14 |
| Human | 15 | 12 |

**Methods**

The enamel-dentine junction (EDJ) surface of each molar was created in .ply format from segmentation of a micro-CT scan of each specimen (Figure S1). MMS reconstructed the missing portions of dentine horns where necessary based on the surrounding preserved portions of the EDJ (although analyses were conducted on both the original and reconstructed models, see below). These reconstructions were completed in Geomagic Wrap 2014.

**
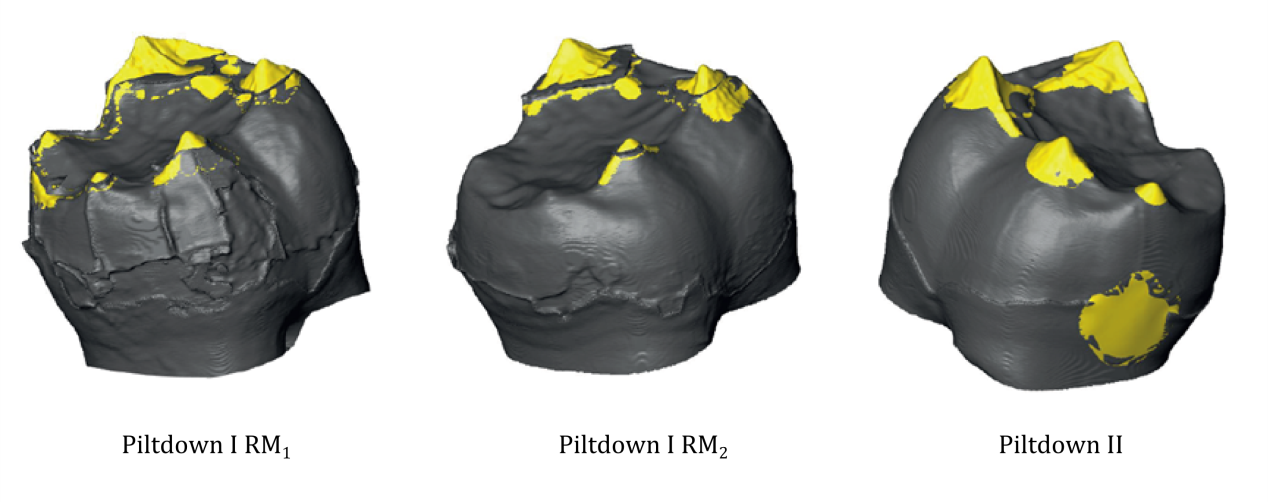
**

Figure S1. Surface models of the three Piltdown molars illustrating their preserved EDJ morphology (grey) and their reconstructed morphology (yellow).

Landmarks were collected at four dentine horns, along the marginal ridge connecting these dentine horns and around the cervix (Figure S2). These landmarks were processed following procedures outlined in Skinner et al. [2, 3] to derive a set of geometrically correspondent landmarks (n = 90) suitable for geometric morphometric analysis [4] . A principal component analysis (PCA) of shape coordinates was conducted to assess EDJ shape variation in the molars of the study sample. Analyses were conducted in both shape and form space (the latter including the natural logarithm of centroid size as an additional variable; [5] ).


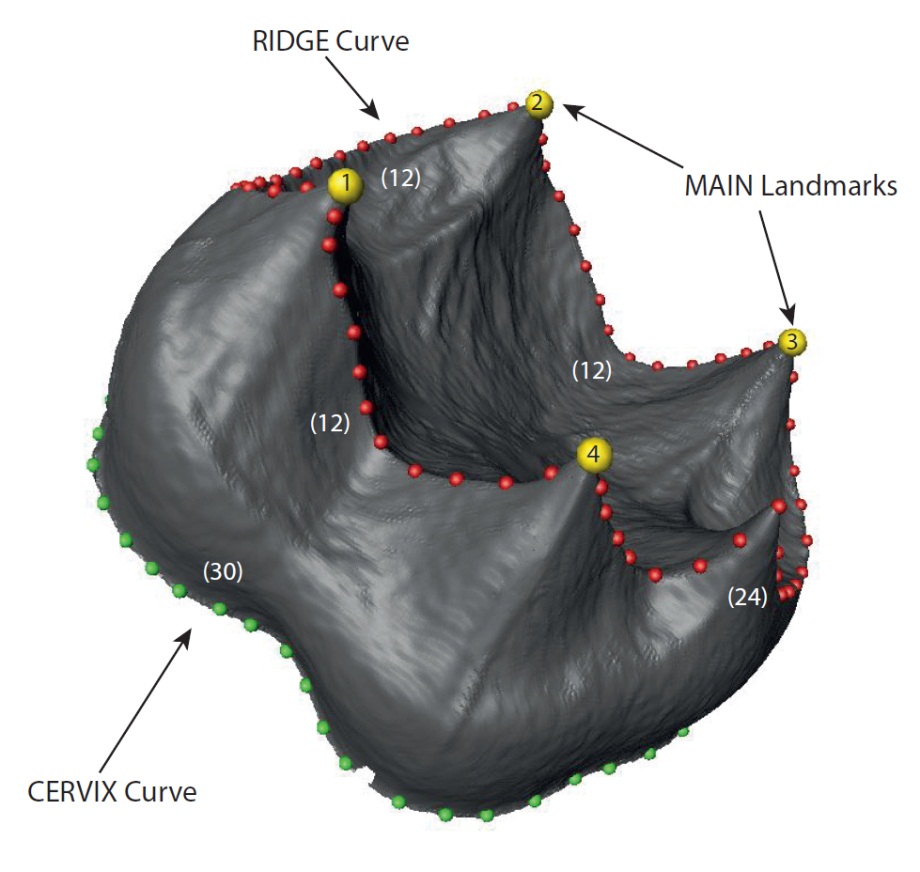


Figure S2. Placement of landmarks at the four dentine horns (yellow), along the marginal ridge (red) and around the cervix (green). For the curve landmark sets, the numbers in brackets represent the number of equidistantly spaced landmarks on that curve (or portion of curve).

A canonical variates analysis (CVA), which generates a linear combination of variables that maximise the ratio of between-group to within-group variation, was used to assess the accuracy with which molars were correctly classified to a taxon (each group assigned equal prior probability). To assess the classification accuracy we used a leave-one-out cross-validation approach in which each specimen was considered to be unknown and then classified to a taxon based on the variation of the remaining specimens [6]. The number of variables used in a CVA has to be less than the number of specimens [7]; however, this is rarely possible in GM analyses of fossil specimens. Therefore, the CVA was calculated using each of 5-20 PC scores, with 20 PCs explaining approximately 95% of shape variation in the PCA of each molar position. Wireframe models were used to visualize and compare the shape of each Piltdown molar to the mean shape of each comparative taxon using a software routine written by Philipp Gunz in Mathematica 9.0.

Table S2 presents the results of the classification accuracy (using the cross-validated CVA) of the comparative sample for each molar and using the following landmark sets: cervix only (referred to as CEJ), EDJ ridge + CEJ (referred to as EDJ full), and EDJ ridge (not including the landmarks locations absent in the non-reconstructed Piltdown specimens) + CEJ (referred to EDJ partial). Overall, classification accuracy of specimens of known taxonomic affiliation is very high, indicating the reliability of these morphological data for assessing the taxonomic affiliation of the Piltdown specimens.

Table S2. Classification accuracy (%) of the comparative sample for each dataset (shape space/form space). For landmark sets, see text above.

| **(Form/shape)** | **M1 CEJ** | **M1 EDJ full** | **M1 EDJ partial** | **M2 EDJ full** | **M2 EDJ partial** | **M2 CEJ** |
| --- | --- | --- | --- | --- | --- | --- |
| Chimpanzee | 87 / 87 | 100 / 100 | 100 / 100 | 100 / 100 | 100 / 100 | 100 / 100 |
| Gorilla | 100 / 100 | 100 / 100 | 100 / 100 | 100 / 100 | 100 / 100 | 100 / 100 |
| Orang-utan | 100 / 100 | 100 / 100 | 100 / 100 | 100 / 100 | 100 / 100 | 86 / 86 |
| Human | 93 / 93 | 100 / 100 | 100 / 100 | 100 / 100 | 100 / 100 | 100 / 100 |

## SI for morphometric analyses

**Radiographs:** Periapical dental radiographs of the Piltdown I canine were made using a portable Aribex NOMAD handheld X-ray device (60Kv) and an EVA Digital #2 sensor.

**Piltdown I canine analyses:**

**Comparative sample**

32 non-human hominoid canines (from Royal College of Surgeons/Elliot Smith Collection, University College London) were selected from specimens where both maxillary and mandibular canines were present and minimally worn (Table S3).

Table S3: Canine sample for comparison with Piltdown I canine.

| **Species** | **Sample size** | **Sex** |
| --- | --- | --- |
| Gorilla | 10 | 4 m / 6 f |
| Chimpanzee | 11 | 5 m / 6 f |
| Orang-utan | 9 | 4 m / 5 f |

**Methods**

*Canine Crown comparisons*

Maximum mesiodistal crown length (MD) and maximum buccolingual crown width (BL) were measured on all actual specimens. MD/BLx100 was calculated and plotted as box and whisker plots split by mandibular/maxillary dentition.

*Canine Pulp chamber and cervical root comparisons*

Radiographs of the Piltdown I material and comparative sample were taken following [8]. Examples of subadult, young adult and older adult non-human hominoid specimens were selected where the ratio of pulp width to root cervix width could be measured. Unfortunately, no similar collection of radiographs exists for the mandibular dentition, so it was assumed that the proportions and ontogenies of great ape upper and lower canines are comparable, and the Piltdown I canine was placed into the upper left canine socket of a small orang-utan cranium and radiographed in the same orientation for consistency.

## SI for DNA analyses

Nine specimens from the sites of Piltdown I and Piltdown II were subjected to DNA extractions (Table S4). Bone powder from the specimens was obtained in a dedicated dead-air cabinet in the aDNA laboratory. All sample/extract manipulation following bone powder collection was carried out in a UV-enabled Class II laminar flow cabinet. To minimise the impact of destructive sampling we did not attempt to de-contaminate the bones prior to sampling. To balance this we suspended bone powder of human origin in 0.5% sodium hypochlorite for 15 minutes followed by thorough rinsing, three times, in molecular grade water (following [9, 10]). DNA was extracted as outlined in [11] with the addition of an extra wash step with PE buffer. If the bone sample appeared particularly stained or darkened in colour, a DNA extraction blank was run every 4 or 8 DNA extractions as a negative control.

Table S4: Piltdown specimens used in DNA analyses with weight of the extraction.

| **Specimen ID** | **context** | **Element** | **Taxonomy** | **Weight extraction** |
| --- | --- | --- | --- | --- |
| E594 | Piltdown I | Mandible | Hominidae | 90 mg |
| E611 | Piltdown I | Canine | Hominidae | 10-20 mg |
| E648 | Piltdown II | PII Molar | Hominidae | 10-20 mg |
| E590 | Piltdown I | Left fronto-parietal | *Homo sapiens* | 70 mg |
| E591 | Piltdown I | Left temporal | *Homo sapiens* | 80 mg |
| E647 | Piltdown II | Occipital | *Homo sapiens* | 110 mg |
| E592 | Piltdown I | Right parietal | *Homo sapiens* | 50 mg |
| E593 | Piltdown I | Occipital | *Homo sapiens* | 50 mg |
| E646 | Piltdown II | Frontal fragment | *Homo sapiens* | 50 mg |
|  |  |  |  |  |

**PCR amplification**

*Non-human DNA*

We designed PCR primers targeting 183bp (in two overlapping 103bp fragments, including primers) of the mitochondrial d-loop that distinguish the Sumatran orang-utan (*Pongo abelii*) from the Bornean orang-utan (*P. pygmaeus*) whilst capturing the major phylogenetic and phylogeographic variation of modern wild orang-utans [12, 13]. Reference data were collected from GenBank and previously published literature [13-16] (Table S5) and aligned in Geneious R7 (Biomatters) using MAFFT v7.017 [17]. Primers were designed in Primer 3 [18, 19] and verified for specificity using NCBI blast (http://blast.ncbi.nlm.nih.gov/). We also retrieved primers from the literature [20, 21], targeting short d-loop fragments specific to chimpanzees and gorillas, which we used to screen the DNA extracts in case the orang-utan primers did not yield PCR products (Table S5).

PCRs were setup using 1U Smart-Taq, 1X Smart-Taq buffer with (NH4)2SO4, and 2.5 mM Smart MgCl2 solution (Naxo Ltd. Tartu, Estonia), 0.5µM each primer, and 200µM dNTPs. To overcome possible inhibition we also added RSA (Sigma-Aldrich) to a final concentration of 0.5 mg/mL. Thermal cycling conditions were 95°C for 15 minutes and 45 cycles of 94°C for 30 seconds, 53°C for 30 seconds and 72°C for 30 seconds, and 72°C for 5 minutes followed by hold at 4°C.

Table S5: PCR Primers

| **Nucleotide position**  **NCBI reference** | **Size bp**  **(incl. primers)** | **Locus** | **Species** | **Reference** | **Tm used here** |
| --- | --- | --- | --- | --- | --- |
| 15,650-15,669 (NC_002083) | 103 | D-loop | *Pongo* sp. | This study | 53°C |
| 15,733-15,752 (NC_002083) |  | D-loop | *Pongo* sp. | This study | 53°C |
| 15,570-15,590 (NC_002083) | 103 | D-loop | *Pongo* sp. | This study | 53°C |
| 15,655-15,672 (NC_002083) |  | D-loop | *Pongo* sp. | This study | 53°C |
| 15,634-16,656 (NC_001643) | 137 | D-loop | *Pan* sp. | Mohandesan et al. 2012 | 53°C |
| 15,749-15,770 (NC_001643) |  | D-loop | *Pan* sp. | Mohandesan et al. 2012 | 53°C |
| 15,658-15,681 (NC_011120) | 118 | D-loop | *Gorilla* sp. | Hofreiter et al. 2003 | 53°C |
| 15,756-15,775 (NC_011120) |  | D-loop | *Gorilla* sp. | Hofreiter et al. 2003 | 53°C |
| 4,542-4,567 (NC_012920) | 80 | ND2 | *Homo* sp. | Malmström et al. 2009 | 60°C |
| 4,595-4,621 (NC_012920) |  | ND2 | *Homo* sp. | Malmström et al. 2009 | 60°C |
|  |  |  |  |  |  |

PCRs were setup using 1U Smart-Taq, 1X Smart-Taq buffer with (NH4)2SO4, and 2.5 mM Smart MgCl2 solution (Naxo Ltd. Tartu, Estonia), 0.5µM each primer, and 200µM dNTPs. To overcome possible inhibition we also added RSA (Sigma-Aldrich) to a final concentration of 0.5 mg/mL. Thermal cycling conditions were 95°C for 15 minutes and 45 cycles of 94°C for 30 seconds, 53°C for 30 seconds and 72°C for 30 seconds, and 72°C for 5 minutes followed by hold at 4°C.

*Human DNA*

In order to identify preserved DNA in human remains from Piltdown I and II, as well as in Dawson’s separate (and also artificially) stained)Barcombe Mills skull (Table 1), we used a previously published quantitative real-time PCR (qPCR) assay targeting an 80bp and 136bp fragment of the mitochondrial coding sequence (ND2) [9, 10]. A standard reference of known concentration was constructed using modern human DNA that was isolated and PCR amplified (anonymised). Modern DNA was extracted using the BuccalAmp kit (Epicentre) following manufacturers recommendations and PCR amplified using primers L4567F- H4595R and L4567F- H4658R [9]. PCRs were set up using 1X KCl Reaction Buffer, 2.5mM MgCl2, 1U Taq DNA polymerase (Bioline), 200µM dNTPs, and 0.5µM of each primer. Cycling conditions were 95°C for 10 min, followed by 35 cycles of 94°C for 30 sec, 58°C for 30 sec, and a final extension at 72°C for 5 min. PCR products were purified using the QIAquick PCR purification kit (Qiagen) and quantified in triplicate using Qubit 2.0 and the Qubit® dsDNA BR Assay Kit (Invitrogen). This value was subsequently converted into the number of corresponding molecules using the Illumina DNA copy number calculator (Illumina), assuming an average molecular weight of 649 daltons/bp for dsDNA. The stock standard was normalized to 108 copies/µL and a series of ten-fold dilutions were made corresponding to 107-101 copies/µL. The standard curves met the following criteria: a correlation between standards at R2>0.99 and that the reaction was efficient (i.e., a doubling of product per cycle in the exponential phase), with values close to 1.0. Data analysis was carried out on the Rotor-Gene 6000 Series Software 1.7.87 (Corbett) following recommendations from Qiagen (www.qiagen.com). At least five standards of known concentration were run in parallel with the Piltdown DNA extracts on a 36-well rotor plate. Standards and DNA extracts were run in duplicate (apart from the positive control which was run in a single well).

Quantitative PCRs were setup using the Fast Rotor-Gene SYBR green kit (Qiagen) on a real-time PCR cycler (Rotor-Gene 6000, Qiagen) following manufacturer recommendations and protocols. Five microliters of DNA extract were used in each PCR reaction. Thermocycling conditions followed Qiagen’s protocol for the Fast Rotor-Gene SYBR Green kit in which denaturation and annealing is fused into one step for 10 sec at 60°C and elongation at 72°C for 5 sec, following a heat activation step at 95°C for 5 minutes. PCRs were run for 45 cycles and primer specificity was assessed using a post-PCR melt curve analysis. Data were analysed by setting a manual threshold, arbitrarily, at 0.5.

*Sequencing and data analysis*

Two independently replicated PCR products/fragments/specimens were purified using ExoSAP-IT (Affymetrix) and pooled in equimolar ratio. The pooled and purified products were built into barcoded Illumina sequencing libraries using the TruSeq Nano DNA sample prep kit v2 (Illumina) following manufacturers recommendations (excluding shearing) by the Sequencing Facility at the NHM. Sequencing was carried out on the Illumina MiSeq platform at ca. 1% of a full run. Sequences were de-multiplexed on the platform and exported in FASTQ format. We processed the sequences in Geneious R7 (Biomatters) first by trimming regions with ≥5% chance error base calls, and secondly by keeping sequences with no more than two bases at quality ≤30. Remaining bases with a quality of ≤30 were masked when calling consensus sequences.

*Phylogenetic analysis*

We aligned the Piltdown DNA sequences with 79 previously published orang-utan sequences (Table S6) using MAFFT v7.017 [17] in Geneious R7 (Biomatters). Haplotypes were collapsed and constructed into a Maximum Likelihood (ML) tree in PhyML (Guindon and Gascuel, 2003) (Figure 4) using the HKY85 model of nucleotide substitution, fixed transition/transversion ratio, estimated proportion of invariable sites, 4 substitution rate categories, and estimated gamma distribution. We performed 100 bootstrap replicates and optimized topology/length under the NNI (default) topology search.

Table S6: Modern orang-utan reference samples.

| **ID** | **GenBank** | **Publication** | **Original ID** | **Haplotype** | **Subspecies** | **Geographic origin** | **Sub-region** | **Status** | **No's** |
| --- | --- | --- | --- | --- | --- | --- | --- | --- | --- |
| 1 | AJ391115 | Warren et al. 2001 | OU-PU1 | 32 | *Pongo pygmaeus** | Wanariset, confiscated in East Kalimantan | E | R |  |
| 2 | AJ391127 | Warren et al. 2001 | OU-SU45 | 33 | *Pongo abelii* | Bohorok, North Sumatra | Sum | R |  |
| 3 | AJ391130 | Warren et al. 2001 | OU-SUHS | 29 | *Pongo abelii* | Known Sumatran origin, Perth Zoo | Sum | C |  |
| 4 | AJ391131 | Warren et al. 2001 | OU-SUUT | 30 | *Pongo abelii* | Known Sumatran origin, Perth Zoo | Sum | C |  |
| 5 | AJ391128 | Warren et al. 2001 | OU-SU46 | 31 | *Pongo abelii* | Bohorok, North Sumatra | Sum | R |  |
| 6 | AJ391129 | Warren et al. 2001 | OU-SU76 | 31 | *Pongo abelii* | Bohorok, North Sumatra | Sum | R |  |
| 7 | AJ391122 | Warren et al. 2001 | OU-SEAH | 20 | *Pongo pygmaeus* | Semongok, Sarawak | W | R |  |
| 8 | AJ391104 | Warren et al. 2001 | n/a | 19 | *Pongo pygmaeus* | n/a | n/a | n/a |  |
| 9 | AJ391099 | Warren et al. 2001 | OU-DS29 | 18 | *Pongo pygmaeus* | Leboyan, Danau Sentarum, Northwest Kalimantan | W | W |  |
| 10 | AJ391125 | Warren et al. 2001 | OU-SEUA | 1 | *Pongo pygmaeus* | Semongok, Sarawak | W | R |  |
| 11 | AJ391124 | Warren et al. 2001 | OU-SEOA | 1 | *Pongo pygmaeus* | Semongok, Sarawak | W | R |  |
| 12 | AJ391121 | Warren et al. 2001 | OU-SE8 | 1 | *Pongo pygmaeus* | Semongok, Sarawak | W | R |  |
| 13 | AJ391102 | Warren et al. 2001 | OU-DSME2 | 3 | *Pongo pygmaeus* | Meliau, Danau Sentarum, Northwest Kalimantan | W | W |  |
| 14 | AJ391101 | Warren et al. 2001 | OU-DSME1 | 3 | *Pongo pygmaeus* | Meliau, Danau Sentarum, Northwest Kalimantan | W | W |  |
| 15 | AJ391123 | Warren et al. 2001 | OU-SEBU | 1 | *Pongo pygmaeus* | Semongok, Sarawak | W | R |  |
| 16 | AJ391103 | Warren et al. 2001 | OU-DSRA | 4 | *Pongo pygmaeus* | Radai, Danau Sentarum, Northwest Kalimantan | W | W |  |
| 17 | AJ391100 | Warren et al. 2001 | OU-DSLE1 | 2 | *Pongo pygmaeus* | Leboyan, Danau Sentarum, Northwest Kalimantan | W | W |  |
| 18 | AJ391135 | Warren et al. 2001 | OU-TNK38 | 21 | *Pongo pygmaeus* | Kutai National Park, East Kalimantan | E | W |  |
| 19 | AJ391126 | Warren et al. 2001 | OU-SO69 | 28 | *Pongo pygmaeus* | Wanariset, confiscated in East Kalimantan | n/a | R |  |
| 20 | AJ391098 | Warren et al. 2001 | OU-DO81 | 11 | *Pongo pygmaeus* | Wanariset, confiscated, origin unknown | n/a | R |  |
| 21 | FR717926 | Arora et al. 2010 | SL2 | 11 | *Pongo pygmaeus* | Sungai Landing, Southcentral Borneo | S | W | n/a |
| 22 | FR717922 | Arora et al. 2010 | TU5 | 11 | *Pongo pygmaeus* | Tuanan, Southwest Borneo | S | W | n/a |
| 23 | FR717921 | Arora et al. 2010 | TU4 | 11 | *Pongo pygmaeus* | Tuanan, Southwest Borneo | S | W | n/a |
| 24 | FR717925 | Arora et al. 2010 | SL1 | 12 | *Pongo pygmaeus* | Sungai Landing, Southcentral Borneo | S | W | n/a |
| 25 | FR717927 | Arora et al. 2010 | SL3 | 13 | *Pongo pygmaeus* | Sungai Landing, Southcentral Borneo | S | W | n/a |
| 26 | EU547193 | Jalil et al. 2008 | OU5 | 6 | *Pongo pygmaeus* | Lower Kinabatangan Wildlife Sanctuary | NE | W/R | 3 |
| 27 | EU547201 | Jalil et al. 2008 | OU13 | 7 | *Pongo pygmaeus* | Lower Kinabatangan Wildlife Sanctuary | NE | W/R | 1 |
| 28 | AJ391119 | Warren et al. 2001 | OU-SB70 | 24 | *Pongo pygmaeus* | Lahad Datu, Sabah | NE | W |  |
| 29 | AJ391117 | Warren et al. 2001 | OU-SB57 | 9 | *Pongo pygmaeus* | Sukau, Kinabatangan, Sabah | NE | W |  |
| 30 | EU547191 | Jalil et al. 2008 | OU3 | 10 | *Pongo pygmaeus* | Lower Kinabatangan Wildlife Sanctuary | NE | W/R | 1 |
| 31 | EU547198 | Jalil et al. 2008 | OU10 | 10 | *Pongo pygmaeus* | Lower Kinabatangan Wildlife Sanctuary | NE | W/R | 5 |
| 32 | EU547196 | Jalil et al. 2008 | OU8 | 9 | *Pongo pygmaeus* | Lower Kinabatangan Wildlife Sanctuary | NE | W/R | 1 |
| 33 | EU547195 | Jalil et al. 2008 | OU7 | 9 | *Pongo pygmaeus* | Lower Kinabatangan Wildlife Sanctuary | NE | W/R | 1 |
| 34 | EU547199 | Jalil et al. 2008 | OU11 | 9 | *Pongo pygmaeus* | Lower Kinabatangan Wildlife Sanctuary | NE | W/R | 27 |
| 35 | AJ391141 | Warren et al. 2001 | OU-TP6 | 26 | *Pongo pygmaeus* | Tanjung Harapan, Tanjung Puting, Central Kalimantan | S | W |  |
| 36 | AJ391139 | Warren et al. 2001 | OU-TP15 | 25 | *Pongo pygmaeus* | Tanjung Puting, Central Kalimantan | S | R |  |
| 37 | AJ391132 | Warren et al. 2001 | OU-TE4 | 16 | *Pongo pygmaeus* | Wanariset, confiscated in Central Kalimantan | n/a | R |  |
| 38 | AJ391113 | Warren et al. 2001 | OU-OO42 | 16 | *Pongo pygmaeus* | Confiscated in Semarang, Java, origin unknown | n/a | R |  |
| 39 | AJ391108 | Warren et al. 2001 | OU-GPUN | 16 | *Pongo pygmaeus* | Gunung Palung, Southwest Kalimantan | S | W |  |
| 40 | AJ391107 | Warren et al. 2001 | OU-GPMA | 16 | *Pongo pygmaeus* | Gunung Palung, Southwest Kalimantan | S | W |  |
| 41 | AJ391106 | Warren et al. 2001 | OU-GPJA | 16 | *Pongo pygmaeus* | Gunung Palung, Southwest Kalimantan | S | W |  |
| 42 | AJ391114 | Warren et al. 2001 | OU-PA68 | 16 | *Pongo pygmaeus* | Wanariset, confiscated in Taiwan, origin unknown | n/a | R |  |
| 43 | AJ391105 | Warren et al. 2001 | OU-GP31 | 16 | *Pongo pygmaeus* | Gunung Palung, Southwest Kalimantan | S | W |  |
| 44 | AJ391138 | Warren et al. 2001 | OU-TP14 | 17 | *Pongo pygmaeus* | Tanjung Puting, Central Kalimantan | S | R |  |
| 45 | FR717934 | Arora et al. 2010 | SA8 | 16 | *Pongo pygmaeus* | Sabangau, Southcentral Borneo | S | W | n/a |
| 46 | FR717932 | Arora et al. 2010 | SA6 | 16 | *Pongo pygmaeus* | Sabangau, Southcentral Borneo | S | W | n/a |
| 47 | FR717931 | Arora et al. 2010 | SA5 | 16 | *Pongo pygmaeus* | Sabangau, Southcentral Borneo | S | W | n/a |
| 48 | FR717930 | Arora et al. 2010 | SA3/GP1 | 16 | *Pongo pygmaeus* | Sabangau, Southcentral Borneo | S | W | n/a |
| 49 | FR717933 | Arora et al. 2010 | SA7 | 16 | *Pongo pygmaeus* | Sabangau, Southcentral Borneo | S | W | n/a |
| 50 | FR717929 | Arora et al. 2010 | SA2 | 16 | *Pongo pygmaeus* | Sabangau, Southcentral Borneo | S | W | n/a |
| 51 | FR717928 | Arora et al. 2010 | SA1 | 16 | *Pongo pygmaeus* | Sabangau, Southcentral Borneo | S | W | n/a |
| 52 | FR717924 | Arora et al. 2010 | TU7 | 8 | *Pongo pygmaeus* | Tuanan, Southwest Borneo | S | W | n/a |
| 53 | FR717923 | Arora et al. 2010 | TU6 | 8 | *Pongo pygmaeus* | Tuanan, Southwest Borneo | S | W | n/a |
| 54 | FR717920 | Arora et al. 2010 | TU3 | 8 | *Pongo pygmaeus* | Tuanan, Southwest Borneo | S | W | n/a |
| 55 | FR717918 | Arora et al. 2010 | TU1 | 8 | *Pongo pygmaeus* | Tuanan, Southwest Borneo | S | W | n/a |
| 56 | FR717919 | Arora et al. 2010 | TU2 | 8 | *Pongo pygmaeus* | Tuanan, Southwest Borneo | S | W | n/a |
| 57 | X98472 | Xu and Arnason 1996 | Anna | 5 | *Pongo pygmaeus pygmaeus* | Aalborg Zoo | n/a | C |  |
| 58 | AJ391118 | Warren et al. 2001 | OU-SB60 | 5 | *Pongo pygmaeus* | Kinabatangan, Sabah | NE | W |  |
| 59 | AJ391116 | Warren et al. 2001 | OU-SB372 | 5 | *Pongo pygmaeus* | Sepilok, Sabah | NE | R |  |
| 60 | AJ391120 | Warren et al. 2001 | OU-SB71 | 5 | *Pongo pygmaeus* | Sandakan, Sabah | NE | W |  |
| 61 | FR717935 | Arora et al. 2010 | DV1 | 5 | *Pongo pygmaeus* | Danum Valley, Northeast Borneo | NE | W | n/a |
| 62 | EU547200 | Jalil et al. 2008 | OU12 | 5 | *Pongo pygmaeus* | Lower Kinabatangan Wildlife Sanctuary | NE | W/R | 25 |
| 63 | FR717936 | Arora et al. 2010 | DV2 | 5 | *Pongo pygmaeus* | Danum Valley, Northeast Borneo | NE | W | n/a |
| 64 | EU547197 | Jalil et al. 2008 | OU9 | 5 | *Pongo pygmaeus* | Lower Kinabatangan Wildlife Sanctuary | NE | W/R | 3 |
| 65 | EU547194 | Jalil et al. 2008 | OU6 | 5 | *Pongo pygmaeus* | Lower Kinabatangan Wildlife Sanctuary | NE | W/R | 1 |
| 66 | EU547190 | Jalil et al. 2008 | OU2 | 5 | *Pongo pygmaeus* | Lower Kinabatangan Wildlife Sanctuary | NE | W/R | 1 |
| 67 | EU547189 | Jalil et al. 2008 | OU1 | 5 | *Pongo pygmaeus* | Lower Kinabatangan Wildlife Sanctuary | NE | W/R | 1 |
| 68 | AJ391111 | Warren et al. 2001 | OU-LI64 | 23 | *Pongo pygmaeus* | Wanariset, confiscated in East Kalimantan | n/a | R |  |
| 69 | AJ391133 | Warren et al. 2001 | OU-TNK36 | 15 | *Pongo pygmaeus* | Kutai National Park, East Kalimantan | E | W |  |
| 70 | AJ391137 | Warren et al. 2001 | OU-TNK41 | 15 | *Pongo pygmaeus* | Kutai National Park, East Kalimantan | E | W |  |
| 71 | AJ391134 | Warren et al. 2001 | OU-TNK37 | 15 | *Pongo pygmaeus* | Kutai National Park, East Kalimantan | E | W |  |
| 72 | AJ391136 | Warren et al. 2001 | OU-TNK39 | 15 | *Pongo pygmaeus* | Kutai National Park, East Kalimantan | E | W |  |
| 73 | AJ391110 | Warren et al. 2001 | OU-KPC | 14 | *Pongo pygmaeus* | Sangatta, East Kalimantan | E | W |  |
| 74 | AJ391109 | Warren et al. 2001 | OU-KA1 | 14 | *Pongo pygmaeus* | Sangatta, East Kalimantan | E | W |  |
| 75 | AJ391112 | Warren et al. 2001 | OU-MU1 | 22 | *Pongo pygmaeus* | Wanariset, confiscated in East Kalimantan | n/a | R |  |
| 76 | X97709 | Xu and Arnason 1996 | Dennis | 14 | *Pongo pygmaeus pygmaeus* | Aalborg Zoo | n/a | C |  |
| 77 | AJ391140 | Warren et al. 2001 | OU-TP24 | 27 | *Pongo pygmaeus* | Tanjung Puting, Southwest Kalimantan | S | W |  |
| 78 | EU547192 | Jalil et al. 2008 | OU4 | 6 | *Pongo pygmaeus* | Lower Kinabatangan Wildlife Sanctuary | NE | W/R | 2 |
| *Replaced GenBank sequence groups with *P. abelii* while original sequence groups with *P. pygmaeus* | | | | | | |  |  |  |
|  | | |  |  |  |  |  |  |  |

## SI for dating analyses

All specimens were processed for radiocarbon dating at the Oxford Radiocarbon Accelerator Unit (ORAU), University of Oxford. Collagen extraction was undertaken using the methods outlined by Brock et al. [22]. Combusted collagen samples weighing ~4-5 mg were analysed using an EA-CF-IRMS system (SERCON 20-22 IRMS linked with a SERCON GSL elemental analyser) with a He carrier gas operating in continuous flow mode. This approach enables the measurement of δ15N and δ13C, nitrogen and carbon content and calculation of C:N atomic ratios. δ13C values for radiocarbon measurements cited in this section are reported with reference to VPDB [23]. The CO2 was cryogenically distilled and then graphitized using published methods, before AMS dating.

As part of the process of radiocarbon dating carbon and nitrogen stable isotopes are measured on the extracted collagen. Based on replicate analyses of international and laboratory standards, precision is ±0.2‰ for δ^13^C and ±0.3‰ δ^15^N.

## SI for staining analyses

The XRF analyses on the Piltdown specimens (ape, human and associated fauna) show various elements on their surfaces, notably iron and on some specimens, chromium, but their proportions vary from specimen to specimen. Zinc, barium and manganese and other elements also appear occasionally.

This distinctive putty was discernible in a number of specimens. XRF spectroscopy was unable to identify the precise composition of the putty, but the elemental make-up was consistent throughout the measured areas (rich in zinc and zirconium) (SI), matching the putty observed in micro-CT scans across specimens.

However, in the CT slices sharp margins between the putty and original frontoparietal bone were apparent (Figure 6), and XRF spectroscopy confirms similar elemental properties of this material to the putty that is also observed in the heavily fractured first molar in the mandible (Figure 6).

## SI for Focus variation microscopy

A Focus Variation Microscope (FVM), the Alicona Infinite Focus optical surface measurement system, based at the NHM was used to collect 3D micro-morphological models of surface modifications on the teeth [24, 25]. Images were captured using a 10x lens (magnification = 91.44 x; vertical (z) resolution = 379 nm, lateral (l) resolution = 3.91 µm) and a 20x lens for details (magnification = 182.88x; z resolution = 182 nm; l resolution = 2.93 µm).

References

1. Johnson T.R., Krauss B., Sedlmair M., Grasruck M., Bruder H., Morhard D., Fink C., Weckbach S., Lenhard M., Schmidt B. 2007 Material differentiation by dual energy CT: initial experience. *European radiology* **17**(6), 1510-1517.

2. Skinner M.M., Gunz P., Wood B.A., Boesch C., Hublin J.J. 2009 Discrimination of extant *Pan* species and subspecies using the enamel–dentine junction morphology of lower molars. *American journal of physical anthropology* **140**(2), 234-243.

3. Skinner M.M., Gunz P., Wood B.A., Hublin J.J. 2008 Enamel-dentine junction (EDJ) morphology distinguishes the lower molars of *Australopithecus africanus* and *Paranthropus robustus*. *Journal of human evolution* **55**(6), 979-988.

4. Gunz P., Mitteroecker P. 2013 Semilandmarks: a method for quantifying curves and surfaces. *Hystrix, the Italian Journal of Mammalogy* **24**(1), 103-109.

5. Mitteroecker P., Gunz P., Windhager S., Schaefer K. 2013 A brief review of shape, form, and allometry in geometric morphometrics, with applications to human facial morphology. *Hystrix, the Italian Journal of Mammalogy* **24**(1), 59-66.

6. Kovarovic K., Aiello L.C., Cardini A., Lockwood C.A. 2011 Discriminant function analyses in archaeology: are classification rates too good to be true? *Journal of Archaeological Science* **38**(11), 3006-3018.

7. Mitteroecker P., Bookstein F. 2011 Linear discrimination, ordination, and the visualization of selection gradients in modern morphometrics. *Evolutionary Biology* **38**(1), 100-114.

8. Dean M.C., Wood B.A. 2003 A digital radiographic atlas of the great ape skull and dentition. In *Digital Archives of Human Paleobiology 3* (eds. Bondioli L., Machchiarelli R.). Milano, Italy, ADS Solutions.

9. Malmström H., Gilbert M.T.P., Thomas M.G., Brandström M., Storå J., Molnar P., Andersen P.K., Bendixen C., Holmlund G., Götherström A. 2009 Ancient DNA reveals lack of continuity between neolithic hunter-gatherers and contemporary Scandinavians. *Current Biology* **19**(20), 1758-1762.

10. Skoglund P., Malmström H., Raghavan M., Storå J., Hall P., Willerslev E., Gilbert M.T.P., Götherström A., Jakobsson M. 2012 Origins and genetic legacy of Neolithic farmers and hunter-gatherers in Europe. *Science* **336**(6080), 466-469.

11. Flink L.G., Allen R., Barnett R., Malmström H., Peters J., Eriksson J., Andersson L., Dobney K., Larson G. 2014 Establishing the validity of domestication genes using DNA from ancient chickens. *Proceedings of the National Academy of Sciences* **111**(17), 6184-6189.

12. Arora N., Nater A., van Schaik C.P., Willems E.P., van Noordwijk M.A., Goossens B., Morf N., Bastian M., Knott C., Morrogh-Bernard H. 2010 Effects of Pleistocene glaciations and rivers on the population structure of Bornean orangutans (*Pongo pygmaeus*). *Proceedings of the National Academy of Sciences* **107**(50), 21376-21381.

13. Warren K.S., Verschoor E.J., Langenhuijzen S., Swan R.A., Vigilant L., Heeney J.L. 2001 Speciation and intrasubspecific variation of Bornean orangutans, Pongo pygmaeus pygmaeus. *Molecular Biology and Evolution* **18**(4), 472-480.

14. Arora N., Nater A., van Schaik C.P., Willems E.P., van Noordwijk M.A., Goossens B., Morf N., Bastian M., Knott C., Morrogh-Bernard H., et al. 2010 Effects of Pleistocene glaciations and rivers on the population structure of Bornean orangutans (*Pongo pygmaeus*). *Proceedings of the National Academy of Sciences* **107**(50), 21376-21381. (doi:10.1073/pnas.1010169107).

15. Jalil M.F., Cable J., Sinyor J., LACKMAN‐ANCRENAZ I., Ancrenaz M., Bruford M.W., Goossens B. 2008 Riverine effects on mitochondrial structure of Bornean orang‐utans (Pongo pygmaeus) at two spatial scales. *Molecular Ecology* **17**(12), 2898-2909.

16. Xu X., Arnason U. 1996 The mitochondrial DNA molecule of Sumatran orangutan and a molecular proposal for two (Bornean and Sumatran) species of orangutan. *Journal of Molecular Evolution* **43**(5), 431-437.

17. Katoh K., Misawa K., Kuma K.i., Miyata T. 2002 MAFFT: a novel method for rapid multiple sequence alignment based on fast Fourier transform. *Nucleic acids research* **30**(14), 3059-3066.

18. Koressaar T., Remm M. 2007 Enhancements and modifications of primer design program Primer3. *Bioinformatics* **23**(10), 1289-1291. (doi:10.1093/bioinformatics/btm091).

19. Untergasser A., Cutcutache I., Koressaar T., Ye J., Faircloth B.C., Remm M., Rozen S.G. 2012 Primer3—new capabilities and interfaces. *Nucleic acids research* **40**(15), e115-e115.

20. Hofreiter M., Siedel H., Van Neer W., Vigilant L. 2003 Mitochondrial DNA sequence from an enigmatic gorilla population (Gorilla gorilla uellensis). *American journal of physical anthropology* **121**(4), 361-368.

21. Mohandesan E., Prost S., Hofreiter M. 2012 Case Study: Using a Nondestructive DNA Extraction Method to Generate mtDNA Sequences from Historical Chimpanzee Specimens. *Ancient DNA: Methods and Protocols*, 101-110.

22. Brock F., Higham T., Ditchfield P., Bronk Ramsey C. 2010 Current pretreatment methods for AMS radiocarbon dating at the Oxford Radiocarbon Accelerator Unit (ORAU). *Radiocarbon* **52**(1), 103-112.

23. Coplen T.B. 1995 Discontinuance of SMOW and PDB. *Nature* **373**, 285.

24. Bello S.M., Soligo C. 2008 A new method for the quantitative analysis of cutmark micromorphology. *Journal of Archaeological Science* **35**(6), 1542-1552.

25. Bello S.M., Verveniotou E., Cornish L., Parfitt S.A. 2011 3‐dimensional microscope analysis of bone and tooth surface modifications: comparisons of fossil specimens and replicas. *Scanning* **33**(5), 316-324.
