# Supplementary material for: New genetic and morphological evidence suggests a single hoaxer created ‘Piltdown man’
Source: R Soc Open Sci. 2016 Aug 10;3(8):160328. doi: 10.1098/rsos.160328 (PMC5108962; doi:10.1098/rsos.160328)
Supplement: Press statement.docx [file rsos160328supp2.docx]

Press statement Short and Long

***New Piltdown study suggests even more strongly that Charles Dawson was the forger***

A team of scientists restudied the Piltdown 'fossils' using the latest scientific methods (DNA analyses, high-precision measurements, spectroscopy and virtual anthropology) and show that it is highly likely a single orang-utan specimen and at least two humans were used to create the fakes. A consistent modus operandi in modification links the studied specimens from Piltdown I and Piltdown II to a single forger - almost certainly Charles Dawson. Whether Dawson acted alone is uncertain but his hunger for acclaim may have driven him to risk his reputation and misdirect the course of anthropology for decades.

***New Piltdown study suggests even more strongly that Charles Dawson was the forger***

Between 1912 and 1914 palaeontologist Arthur Smith Woodward and amateur antiquarian Charles Dawson announced the discovery of fossils from gravels at Piltdown site I (Sussex), which supposedly represented a new evolutionary link between apes and humans: *Eoanthropus dawsoni* (Dawson’s Dawn Man). Before he died on 10^th^ August 1916, Dawson claimed to have discovered further evidence of 'Piltdown Man' at a second site (Piltdown II). Between 1953 and 1955, the remains were shown to have been fraudulently modified to appear ancient and were planted in the sites.

A team of scientists have restudied the Piltdown 'fossils' using the latest scientific methods (DNA analyses, high-precision measurements, spectroscopy and virtual anthropology) and show that it is highly likely that a single orang-utan specimen and at least two human specimens were used to create the fakes. A consistent *modus operandi* in modification links the studied specimens from Piltdown I and Piltdown II to a single forger - almost certainly Charles Dawson. Whether Dawson acted alone is uncertain but his hunger for acclaim may have driven him to risk his reputation and misdirect the course of anthropology for decades.
